# 598. Clinical Characteristics and Characterization of *Hafnia Alvei* Infections in Cancer Patients

**DOI:** 10.1093/ofid/ofad500.665

**Published:** 2023-11-27

**Authors:** Keval Thakkar, Heeya Shah, Matthew Larsen, Sadaf Aslam, John Greene

**Affiliations:** Moffitt Cancer Centre, Arlington, Virginia; Moffitt Cancer Centre, Arlington, Virginia; Univesity of South Florida, Tampa, Florida; University of South Florida Morsani College of Medicine, Tampa, Florida; Moffitt Cancer Center, Tampa, FL

## Abstract

**Background:**

There have been documented cases, although rare, of clinically significant infections caused by *Hafnia alvei* leading to infections such as bacteremia, pneumonia, UTI, meningitis, and empyema. *H. alvei* infections can be community-acquired or nosocomial. The published data for *H. alvei* in cancer patients is limited. The aim of this study was to characterize *H. alvei* infections including patient characteristics, antimicrobial susceptibility patterns, location of isolation, and microbial characteristics of isolates in cancer patients.

**Methods:**

A retrospective cohort study was performed to review records of all consecutive patients with a positive *H. alvei* culture during the past 7 years. Variables included patient’s age, sex, underlying malignancy, neutrophil count, location and duration of infection, antimicrobial susceptibility patterns, hospital stay, co-isolates, and outcomes.

**Results:**

A total of 15 patients with positive *H. alvei* cultures were identified at the Moffitt Cancer Center. There were 8 (53%) female and 7 (47%) male patients. The mean patient age was 65.7 ±15.8 yrs. All patients had underlying malignancies, most common type was solid organ malignancy in 11 patients (73%), and hematologic malignancies in 4 patients (27%). Two patients (13%) were neutropenic at the time of positive culture. *H. alvei* isolates were first identified in patients at a median of 8 days and an average of 11 days after admission to the hospital. Of the 15 isolates, 8 (53%) were monomicrobial and 7 (47%) were polymicrobial cultures (Table 1). Co-isolates and susceptibilities are shown in the Table 2 and 3.

Table 1

**Figure:**
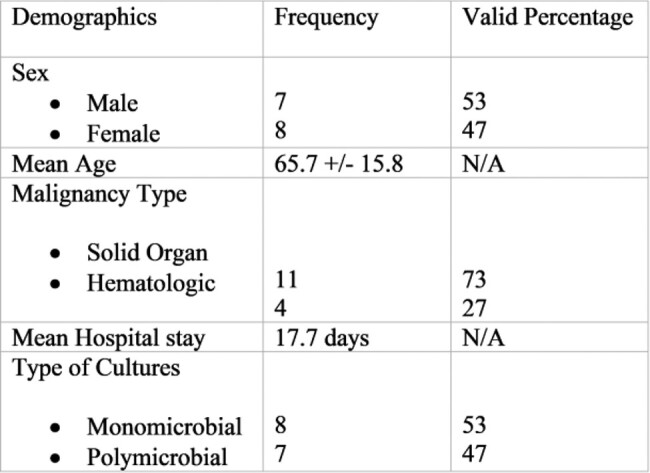

Demographic data for all identified patients

Table 2

**Figure:**
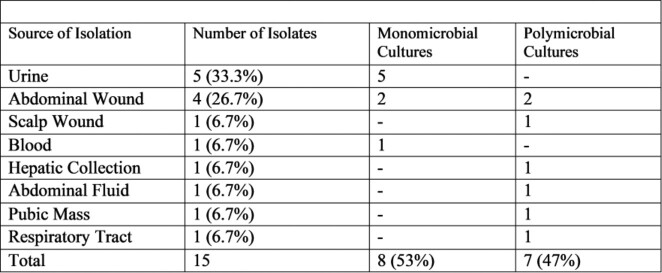

Characteristics of H. alvei isolations

Table 3

**Figure:**
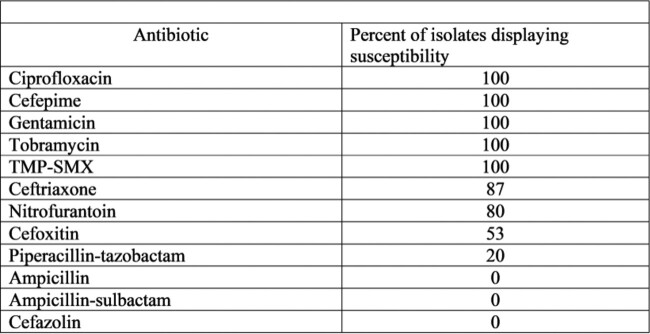

H. alvei antibiotic susceptibility report

**Conclusion:**

This study demonstrated that *H. alvei* is a truly rare pathogenic organism with only 15 documented cases of isolation over an 80-month time period at an academic cancer center. In patients with underlying malignancies, the organism tends to have a predilection for both the urinary tract and abdominal wounds, and can be isolated from pure cultures in the urine and with co-isolates in all other locations. Our study showed that resistance with *H. alvei* is usually with ampicillin and most of our isolates were resistant to cefazolin and cefoxitin. When infection does occur, isolates tend to be susceptible to standard antibiotics such as aminoglycosides and ciprofloxacin.

**Disclosures:**

**All Authors**: No reported disclosures

